# Whole-Exome Sequencing Enables the Diagnosis of Variant-Type Xeroderma Pigmentosum

**DOI:** 10.3389/fgene.2019.00495

**Published:** 2019-05-24

**Authors:** Xiaokai Fang, Yonghu Sun

**Affiliations:** Shandong Provincial Hospital of Dermatology, Shandong University, Jinan, China

**Keywords:** whole-exome sequencing, xeroderma pigmentosum (XP), DNA polymerase eta (*POLH*) gene, novel mutation, psoriasis

## Abstract

**Background:**

Xeroderma pigmentosum (XP) is a rare autosomal, recessive, inherited disease. XP patients exhibit high sensitivity to sunlight and increased incidence of skin cancer. The different XP subtypes, which are caused by mutations of eight distinct genes, show some specific clinical manifestations. XP variant (XPV) is caused by mutations in the gene encoding DNA polymerase eta (*POLH*).

**Case Presentation:**

We report a family that included two XP patients whose parents were first cousins. The proband is a 36-year-old male who developed a large number of pigmented freckle-like lesions starting at 4 years of age; later, he displayed typical psoriasis manifestation, abnormal renal function and hyperglycaemia. He was suspected as suffering from dyschromatosis symmetrica hereditaria (DSH), but negative results were obtained in candidate gene analyses. Whole-exome sequencing was performed in four subjects, including the two patients and two controls, and a new pathogenic homozygous nonsense mutation (c.353dupA, p. Y118_V119delinsX) of the *POLH* gene, which was identified in all nine family members by Sanger sequencing, was detected in the patients.

**Conclusion:**

A novel XPV pathogenic homozygous nonsense mutation in the *POLH* gene was identified. Our case proves that next-generation sequencing is an effective method for the rapid diagnosis and determination of XP genetic etiology.

## Background

Xeroderma pigmentosum (XP) is a rare autosomal recessive disorder resulting from deficiency in base excision repair caused by single-nucleotide mutations, especially in skin exposed to sunlight (Okamura et al., 2015). XP is classified into eight subtypes. Patients with XP show light sensitivity and skin pigmental changes in sun-exposed areas and have a higher incidence of neurological abnormalities, skin cancer, and other tumors (Ben Rekaya et al., 2018). Although the different XP subtypes present some specific clinical manifestations, the conditions are usually difficult to diagnose, and subtypes are defined only based on clinical manifestations if patients display mild phenotype or early-stage uncharacteristic manifestations.

The gene responsible for each type of XP has been identified. Most of the eight distinct genes encode proteins associated with the nucleotide excision repair (NER) function (Lehmann et al., 2011). The XP variant (XPV) type is the only variant that does not involve mutation in NER pathway components and instead results from mutations in the XPV gene. XPV is also named the DNA polymerase eta (*POLH*) gene and encodes the Y-DNA polymerase that participates in the repair of damaged DNA (Ohmori et al., 2001). *POLH* mutations weaken DNA of replication under exposure to ultraviolet light (Cordonnier et al., 1999).

In this study, we performed whole-exome sequencing of an XP family, which had been suspected as having dyschromatosis symmetrica hereditaria (DSH), and a novel XP pathogenic homozygous nonsense mutation (c.353dupA, p. Y118_V119delinsX) was identified in the *POLH* gene (NM_006502). Our case proves that next-generation sequencing (NGS) is an effective method for the rapid diagnosis and determination of XP genetic etiology.

## Case Presentation

**FIGURE 1:**
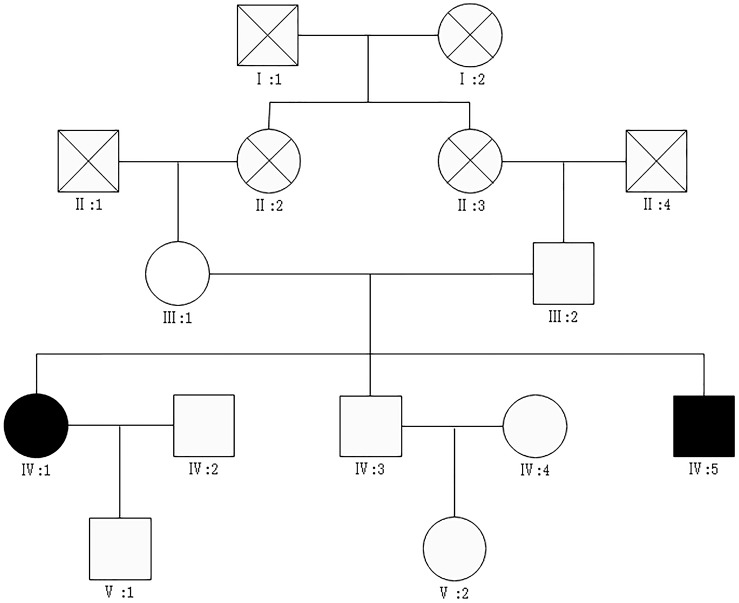
The pedigrees of the patients. 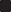 affected male, 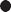 affected female, 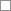 unaffected male, 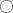 unaffected female, X mark: deceased.

**FIGURE 2:**
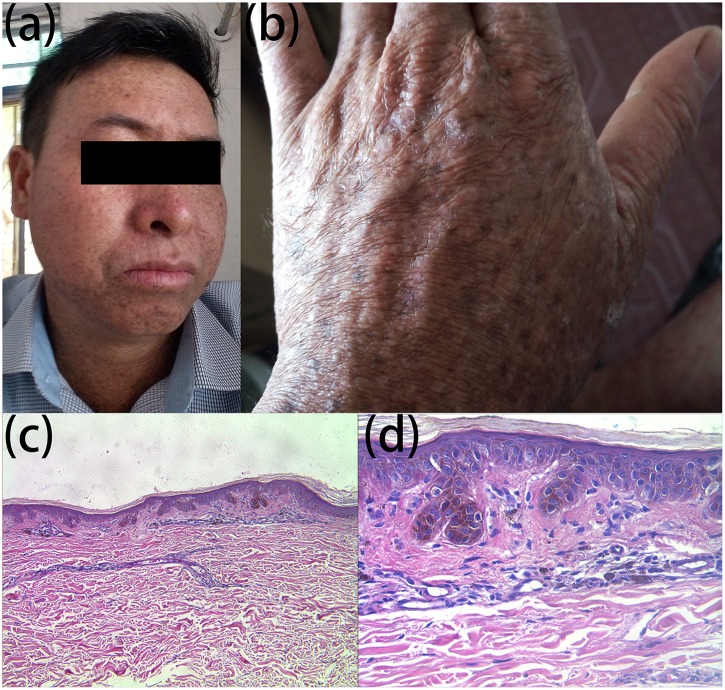
Clinical and histopathological appearance of hyperpigmentation. Hyperpigmented lesions on the face **(a)** and dorsal hand **(b)**; melanin pigmentation in the basal layer and extension of the furcella into the dermis in a bud shape **(c)** HE stain, × 100; **(d)** HE stain, × 400.

**FIGURE 3:**
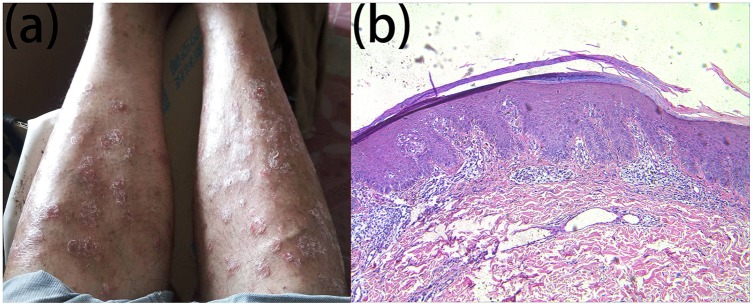
Clinical and histopathological appearance of psoriasis. Lesions on the lower limbs **(a)** continuous parakeratosis and Munro microabscesses in the epidermal layer, dilated superficial vessels, and infiltration of a few lymphocytes in the dermis layer **(b)** HE stain, × 100.

We report a family that included two patients whose parents are first cousins. The proband (Figure 1: IV:5) is a 36-year-old male who developed many pigmented freckle-like patches in the skin starting at 4 years of age. These changes were particularly located in UV-exposed areas, such as the face (Figure 2a), upper thorax, upper limbs, dorsal hands (Figure 2b), feet and legs, and gradually worsened before 18 years of age, after which the condition stabilized. Over the entire time period, sunlight aggravated the disease. The palms, planta pedis, scalp, mucous membranes and nervous system were not affected. Histopathological examination of the skin lesions was performed and revealed melanin pigmentation in the basal layer and extension of the furcella into the dermis with a bud shape (Figure 2c,d). The patient presented a typical psoriasis manifestation at 35 years of age. Irregular geographic erythema appeared mainly in the extremities and gradually progressed. The skin lesions were covered with thick scales that had characteristics of the wax droplet phenomenon, the membrane phenomenon and dotty hemorrhage (Figure 3a). Upon histopathological examination of the skin lesions, the epidermal layer showed continuous parakeratosis and Munro’s microabscesses. The dermis layer exhibited dilated superficial vessels and infiltration of a few lymphocytes (Figure 3b). Abnormal renal function was also found, with elevated levels of creatinine 152.2 μmol/L (normal 44–132.6 μmol/L) and uric acid 552 μmol/L (normal 44–132.6 μmol/L), at the age of 35. At 36 years of age, the patient was found to have hyperglycaemia, with elevated fasting blood glucose in multiple tests. No skin tumors were found. The proband’s older sister (IV:1) is a 41-year-old female without any concomitant disease. The sister presented pigmented lesions that were similar to those observed in the proband. Other members in this pedigree were healthy.

This study was approved by the Ethical Committee and was carried out according to the Declaration of Helsinki Principles. Nine people, including the two patients in the family, provided written consent to join the study, including authorization to extract peripheral anticoagulation blood and to publish these case details.

## Description of Laboratory Investigations and Diagnostic Tests

### Whole-Exome Sequencing and Variant Analysis

Genomic DNA was isolated from whole blood samples using a Flexigene^®^ DNA kit based on the manufacturer’s protocol. DNA was available for all nine people in the family, including the two patients.

Whole-exome sequencing was conducted for the patients (IV:1, IV:5) and normal controls (IV:4, V:2). At least 0.6 μg of DNA from each of the subjects was fragmented into 180–280 bp segments using a Covaris S220 sonicator. The Agilent SureSelect Human All Exon V6 kit was employed to enrich, hybridize and capture these fragments following the manufacturer’s specifications. Qubit 2.0 and Agilent 2100 were used for preliminary quantification and detection of the library insert size. qPCR was used for accurate quantification of the effective concentration to ensure the library quality, and an Illumina HiSeq 2000 was utilized for library sequencing.

The Burrows-Wheeler Alignment tool (BWA) was used to match the clean reads without adapters or debased reads to the human reference genome (UCSC hg19)^1^, (Li and Durbin, 2009). Duplicate reads were marked by Picard after the deletion or insertion of nucleotide fragments, and single-nucleotide polymorphisms (SNPs) were identified by Sequence Alignment/Map tools (SAMtools) (Li et al., 2009). The variants were filtered with SNP database 147 (dbSNP147) (Sherry et al., 2001), 1000 Genomes Project (version 2015 August) (Genomes Project et al., 2012), and NHLBI Exome Sequencing Project (ESP) 6500. Sorting Intolerant from Tolerant (SIFT) (Ng and Henikoff, 2003) and Polymorphism Phenotyping version2 (PolyPhen-2) were employed to predict protein function (Adzhubei et al., 2013). Highly suspicious mutations were noted by Annotate Variation (ANNOVAR) software (Wang et al., 2010).

We generated over 40 GB of data by exome sequencing. Among the four samples, there were 77,073,558, 74,946,888, 67,801,236, and 76,068,580 clean data reads obtained, and 99.87, 99.90, 99.84, and 99.93% of the reads were mapped to the human reference genome; the average sequencing depths were 126.61×, 112.91×, 104.50×, and 117.42×, respectively. At least 99.6, 99.3, 97.9, and 99.3% of the targeted exomes were covered and exceeded 10 × depths, which was considered a meaningful standard for identifying SNPs and insertion or deletion mutations. The bases with quality above 99.9% accuracy represented over 90% of the total data. Because XP is an extremely rare genetic disorder, we excluded variants, including those in the 1000 Genomes Project database (Genomes Project et al., 2012), with minor allele frequencies over 0.5%, dbSNP147 (Sherry et al., 2001) with a frequency higher than 0.1%, and NHLBI ESP6500. PolyPhen-2 (Adzhubei et al., 2013) and SIFT (Ng and Henikoff, 2003) were used to predict functional changes due to the candidate mutations as a reference. Ten possible mutation sites, including a novel homozygous nonsense mutation (c.353dupA, p. Y118_V119delinsX) in the *POLH* gene and a homozygous missense mutation (c. T214C, p. S72P) in the *t*-complex-associated-testis-expressed 1 (*TCTE1*) gene (NM_182539) were identified in the two patients. Exome sequencing processing showed that these mutation sites were not present in the normal controls (IV:4, V:2).

### Sanger Sequencing and Functional Prediction

To verify pathogenic mutations, the entire pedigree was subjected to direct Sanger sequencing using an ABI3500 sequencer (Applied Biosystems, Foster City, CA, United States). Primer sequences for candidate pathogenic variants were designed, and segmented primer sequences for the *ADAR* gene were also designed to exclude DSH. The change in a potential pathogenic variant’s function was predicted by MutationTaster (Schwarz et al., 2014). Protein models of DNA polymerase eta were constructed using Swiss-Model (Waterhouse et al., 2018).

**FIGURE 4:**
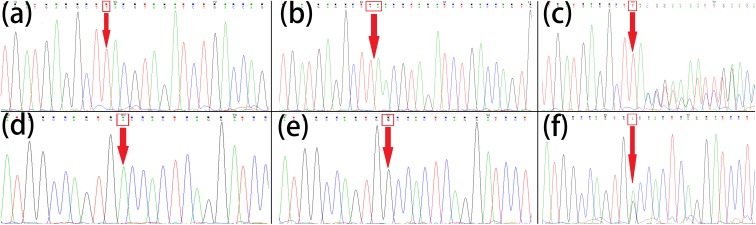
Sanger sequencing of *POLH* and *TCTE1*. Normal control (IV:2, IV:3, IV:4, V2) **(a)**, homozygous mutations (IV:1, IV:5) **(b)** and heterozygous mutations (III:1, III:2, V1) **(c)** of *POLH* (c.353dupA, p. Y118_V119delinsX); normal control (IV:2, IV:3, IV:4, V2) **(d)**, homozygous mutations (IV:1, IV:5) **(e)** and heterozygous mutations (III:1, III:2, V1 **(f)** of *TCTE1* (c. T214C, p. S72P). The mutational bases were arrowed.

Based on Sanger sequencing for the 10 possible mutation sites in all 9 family members, eight sites not fitting the phenotype were excluded (Supplementary Table 1). The *POLH* variant (c.353dupA, p. Y118_V119delinsX) and the *TECE1* variant (c. T214C, p. S72P) were confirmed by Sanger sequencing and identified as being heterozygous in the patients’ normal parents and another family member (III:1, III:2, V1; Figure 4c,f). They were both homozygous variants in the patients (IV:1, IV:5; Figure 4b,e). However, the variants were not found in the rest of the family (IV:2, IV:3, IV:4, V2; Figure 4a,d) and was not reported in the databases searched or previous genome-wide association studies. The homozygous *POLH* variant co-segregating with the disease phenotype in the family was predicted to lead to a change in amino acid sequence and a premature termination codon and to affect protein features and splice site changes, as assessed by MutationTaster (Schwarz et al., 2014); this may be a morbigenous variant in this pedigree. According to Swiss-Model (Waterhouse et al., 2018), the protein model of DNA polymerase eta exhibits different lengths and configurations in the normal controls and patients with mutations in the *POLH* gene, suggesting significant functional deficiency (Supplementary Figure 1). *TCTE1* and *POLH* are both on 6p21.1, and the mutation in *TCTE1* also co-segregated with the phenotype in the entire family.

## Discussion of the Underlying Pathophysiology and the Novelty of the Case

The proband (IV:5) is a 36-year-old male who presented with hyperpigmented macules that appeared in early childhood. His parents are of a consanguineous marriage, and his sister has features similar to his own. We investigated some genetic pigmentation diseases, such as Bloom syndrome, Rothmund-Thomson syndrome, Peutz-Jeghers syndrome, and Cockayne syndrome. Combined with the medical history, DSH was suspected. Indeed, because histopathological examination revealed melanin pigmentation in the basal layer, the diagnosis of DSH seemed reasonable. DSH is caused by a heterozygous mutation in the adenosine deaminase RNA-specific gene (*ADAR*) on chromosome 1q21 and shows a high-penetrance, autosomal dominant inheritance pattern (Xing et al., 2003). Although the genetic model of this family appears to fit recessive inheritance and the patients did not have hypopigmented macules, Sanger sequencing of the *ADAR* gene for five family members, including the two patients (III:1, III:2, IV:1, IV:3 and IV:5), was performed to eliminate possible errors in penetrance and information collection. However, no *ADAR* mutation was detected in this family. Considering that the proband had concomitant symptoms, including psoriasis and multiple organ damage, diagnosis of the disease remained challenging.

Whole-exome sequencing analyses genetic information in a rapid and effective manner, allowing hereditary speculation of complex and monogenic genetic diseases, including skin pathology (Choi et al., 2009). For rare disorders, the application of whole-exome sequencing can minimize mistakes in detecting mutations in hot-spot regions. Specifically, for Mendelian disorders, NGS can be helpful for inferring pathogenesis and exploring new mutations of genes associated with the disease (Yang et al., 2013). Furthermore, this method is very effective for the study of genetic diseases with recessive genetic patterns because it can effectively identify homozygous pathogenic mutations through contrast analysis of the coding sequence between affected and unaffected individuals (Bamshad et al., 2011).

Because the sequencing results for the *ADAR* gene were negative, we utilized high-throughput sequencing to identify the genetic nature of the disease. Through whole-exome and Sanger sequencing, the *POLH* mutation was found to be the pathogenic factor. Combined with the medical history, clinical manifestations, laboratory findings and previous reports (Inui et al., 2008), the diagnosis of XPV caused by a *POLH* mutation was confirmed. This gene encodes certain DNA polymerases that repair DNA damage and inhibit the mutagenicity of UV-induced DNA changes. In addition, *POLH* is proposed to be related to hypermutation in the process of immunoglobulin class switch recombination. To date, approximately one hundred *POLH* mutations have been shown to be associated with XPV pathogenesis (Yuasa et al., 2000; Inui et al., 2008; Opletalova et al., 2014; Karass et al., 2015). In most situations, these mutations result in premature termination codons in mRNA that lead to degradation by the nonsense-mediated decay (NMD) system (Inui et al., 2008). Opletalova et al. (Opletalova et al., 2014) summarized the relationship of phenotype/genotype in 23 XPV patients, showing that the type of missense mutation was clearly related to the clinical severity. For patients with truncating mutations, life-cumulated UV exposure is probably the best predictor of cancer incidence, and it is very necessary to avoid sun exposure. In our case, the variant was a nonsense mutation. The proband showed a stable general condition without any tumor, consistent with another report (Ben Rekaya et al., 2011).

*TCTE1* has not been previously reported as a pathogenic gene. Kwiatkowski et al. (1991) identified highly informative dinucleotide repeat polymorphisms in the *TCTE1* locus that were previously called D6S46. As our proband had some concomitant diseases, such as psoriasis, abnormal renal function and hyperglycaemia, the missense mutation in *TCTE1* may be due to multiple unknown factors or may be meaningless. It is worth mentioning that the homozygous missense *TCTE1* variant completely co-segregated with the disease phenotype in the pedigree and was expected to lead to changes in amino acid sequences and protein features, as well as splice site changes and disease, as assessed by MutationTaster. Both of the mutated genes are located at 6p21.1. Although pure linkage inheritance is highly possible, we cannot exclude the possibility that it is a pathogenic factor related to XPV, a possibility that requires further exploration.

Xeroderma pigmentosum variant patients constitute approximately 20% of all patients with XP. Compared with XP groups A-G, XPV is more benign, presenting with mild skin lesions and low tumor incidence. In addition, symptoms occur late, and patients have a longer life expectancy, with few developing neurological abnormalities (Gratchev et al., 2003). The individuals affected by analogous pigmented diseases and sun sensitivity, such as in XPV, are usually underdiagnosed because patients do not display attentional symptoms, such as apparent damage or carcinomatous degeneration of the light-exposed skin, until a late age. In some cases, skin lesions in XPV patients can vary greatly in the degree of severity (Inui et al., 2008). Overall, molecular diagnosis remains very challenging for these patients (Ben Rekaya et al., 2018).

Xeroderma pigmentosum variant is a cancer-prone syndrome that results in photosensitivity, dermatic and ocular injury, and skin precancerous lesions after sun exposure (Broughton et al., 2002). Tumors of various pathological forms and neurological symptoms are concomitant symptoms. The proband in the current study presented with psoriasis, renal dysfunction and hyperglycaemia. However, the relationship between the primary disease and associated symptoms is unknown. Ezzedine et al. (2008) reported XP accompanied by psoriasis. The pathogenesis of both diseases is completely different, and psoriasis may be another primary disease or may be influenced by XPV. Further investigations are required to determine how these likely irrelevant diseases can coexist. Some previous XP cases exhibited manifestations of end-stage renal disease (Radwan et al., 2015), renal cell carcinoma (Loghin et al., 2016) and diabetes (Das et al., 2018). There are many connections between these conditions, but it is difficult to determine the specificity because renal dysfunction and hyperglycaemia appear to be more similar to the primary disease.

The use of NGS technology is a double-edged sword. Because we cannot accurately diagnose diseases and because known genes have many exons, first-generation sequencing of all exons is difficult and time-consuming (Ben Rekaya et al., 2018). Diseases with unknown mutations have yet to be discovered, and NGS is more efficient than first-generation sequencing approaches, with similar costs (Kleijer et al., 2008). Nonetheless, Ortega-Recalde et al. (2014) reported a bottleneck of downstream bioinformatics analysis of data, and transformation of next-generation technologies into clinical practice remains challenging (Smith et al., 2014). Although some distinct bioinformatics tools have been implemented, much time and high costs have been invested in establishing a reliable NGS platform.

Regardless, our experience proves that whole-exome sequencing is an efficient method that can be used for XP diagnosis and etiology classification in the future. Through various technologies, our understanding of XP will gradually deepen, thus promoting continuous improvement in treating this disease.

## Data Availability

Publicly available datasets were analyzed in this study. This data can be found here: http://genome.ucsc.edu/.

## Ethics Statement

This study has been approved by the Ethical Committee and was conducted according to the Declaration of Helsinki Principles. Nine people, including two patients in the family, provided a written consent form to participate in the study, which included an authorization to extract peripheral anticoagulation blood and to publish these case details.

## Author Contributions

XF made substantial contributions to the conception design, wrote the manuscript, and collected the materials and blood samples of the patients. YS performed data analysis.

## Conflict of Interest Statement

The authors declare that the research was conducted in the absence of any commercial or financial relationships that could be construed as a potential conflict of interest.

## Footnotes

1 http://genome.ucsc.edu/
