# Supplementary material for: Whole-Exome Sequencing Enables the Diagnosis of Variant-Type Xeroderma Pigmentosum
Source: Front Genet. 2019 May 24;10:495. doi: 10.3389/fgene.2019.00495 (PMC6543889; doi:10.3389/fgene.2019.00495)
Supplement: Supplementary file 1 [file Image_1.pdf]

Supplementary Figure 1

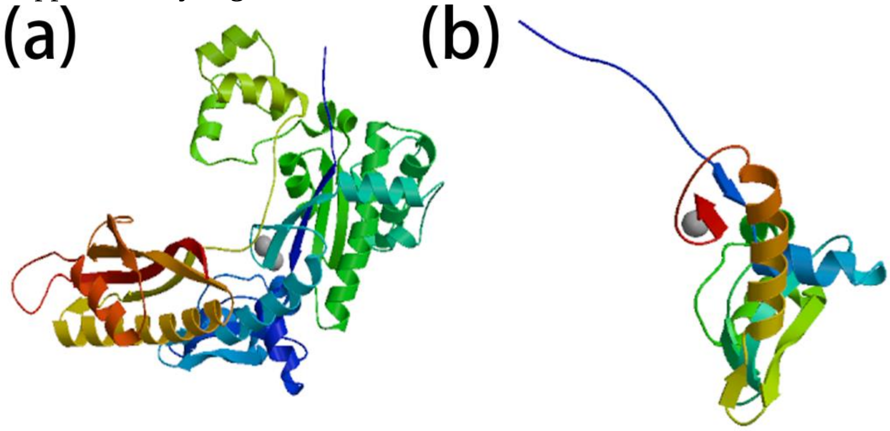

Supplementary Figure 1: Protein model of DNA polymerase eta. Protein models of DNA polymerase eta in normal controls (a) and patients with mutations in the POLH gene (b) show different lengths and configurations.
