# Supplementary material for: Whole-Exome Sequencing Enables the Diagnosis of Variant-Type Xeroderma Pigmentosum
Source: Front Genet. 2019 May 24;10:495. doi: 10.3389/fgene.2019.00495 (PMC6543889; doi:10.3389/fgene.2019.00495)
Supplement: Supplementary file 2 [file Table_1.pdf]

Supplementary Table 1 Possible mutation sites in Whole-exome sequencing and Sanger sequencing results

No.	Coordinate (GRCh37)	Ref	Alt	Gene name	WES+SAS				SAS				
					Patient		Normal		Normal				
					IV1	IV5	IV4	V2	Ⅲ1	Ⅲ2	IV2	IV3	V1
1	6: 43555088	T	TA	*POLH*	1/1	1/1	0/0	0/0	0/1	0/1	0/0	0/0	0/1
2	6: 44255349	A	G	*TCTE1*	1/1	1/1	0/0	0/0	0/1	0/1	0/0	0/0	0/1
3	1:14106396	T	TCCC	*PRDM2*	1/1	1/1	0/0	0/1	0/1	1/1	0/0	1/1	0/1
4	2:24387179	C	CG	*FAM228B*	1/1	1/1	0/0	0/1	0/1	1/1	0/0	0/1	0/1
5	6:160560896	CCTGGTAAG	C	*SLC22A1*	1/1	1/1	0/0	0/1	0/1	0/1	0/0	1/1	0/1
6	7: 82581491	A	ATCC	*PCLO*	1/1	1/1	0/0	0/0	0/1	1/1	0/0	0/1	0/1
7	7: 131195094	GAAAAAA	G	*PODXL*	1/1	1/1	0/0	0/0	0/1	1/1	0/0	0/1	0/1
8	9: 97080926	TCTTGGA	T	*NUTM2F*	1/1	1/1	0/0	0/1	0/1	0/1	0/0	1/1	0/1
9	19:20807178	A	AG	*ZNF626*	1/1	1/1	0/0	0/0	0/1	1/1	0/0	0/1	0/1
10	18: 28662324	C	A	*DSC2*	1/1	1/1	0/0	0/1	0/1	0/1	0/0	1/1	0/1

Ref: reference genome base type; Alt: altered sample genome base type; WES+SAS: Data were obtained by whole-exome sequencing and confirmed by Sanger sequencing. SAS: Data were only obtained by Sanger sequencing; 0: allele is the same as the reference; 1: allele is not the same as the reference.
